# Ketogenic Diet in Alzheimer’s Disease

**DOI:** 10.3390/ijms20163892

**Published:** 2019-08-09

**Authors:** Marta Rusek, Ryszard Pluta, Marzena Ułamek-Kozioł, Stanisław J. Czuczwar

**Affiliations:** 1 Department of Pathophysiology, Medical University of Lublin, 20-090 Lublin, Poland; 2 Department of Dermatology, Venereology and Pediatric Dermatology, Laboratory for Immunology of Skin Diseases, Medical University of Lublin, 20-080 Lublin, Poland; 3 Laboratory of Ischemic and Neurodegenerative Brain Research, Mossakowski Medical Research Centre, Polish Academy of Sciences, 02-106 Warsaw, Poland; 4 First Department of Neurology, Institute of Psychiatry and Neurology, 02-957 Warsaw, Poland

**Keywords:** Alzheimer’s disease, ketogenic diet, amyloid, tau protein, neuroinflammation, dementia, ketone bodies therapy

## Abstract

At present, the prevalence of Alzheimer’s disease, a devastating neurodegenerative disorder, is increasing. Although the mechanism of the underlying pathology is not fully uncovered, in the last years, there has been significant progress in its understanding. This includes: Progressive deposition of amyloid β-peptides in amyloid plaques and hyperphosphorylated tau protein in intracellular as neurofibrillary tangles; neuronal loss; and impaired glucose metabolism. Due to a lack of effective prevention and treatment strategy, emerging evidence suggests that dietary and metabolic interventions could potentially target these issues. The ketogenic diet is a very high-fat, low-carbohydrate diet, which has a fasting-like effect bringing the body into a state of ketosis. The presence of ketone bodies has a neuroprotective impact on aging brain cells. Moreover, their production may enhance mitochondrial function, reduce the expression of inflammatory and apoptotic mediators. Thus, it has gained interest as a potential therapy for neurodegenerative disorders like Alzheimer’s disease. This review aims to examine the role of the ketogenic diet in Alzheimer’s disease progression and to outline specific aspects of the nutritional profile providing a rationale for the implementation of dietary interventions as a therapeutic strategy for Alzheimer’s disease.

## 1. Introduction

Alzheimer’s disease (AD) is the most significant cause of dementia that affects around 50 million people worldwide [1]. It is a heterogeneous and multifactorial disorder, characterized by cognitive impairment with a progressive decline in memory, disorientation, impaired self-care, and personality changes [2,3]. The most common symptom present at the beginning of AD is associated with short term memory deficit, which affects daily activities [3]. Cognitive deficits, resulting from the loss of neurons, are susceptible to neurofibrillary degeneration located in the limbic system, subcortical structures, archicortex and neocortex, and progressive synaptic dysfunction [4]. Pathologically, AD involves progressive deposition of amyloid β-peptide (Aβ) as amyloid plaques, hyperphosphorylated tau protein intracellularly as neurofibrillary tangles (NFTs) and neuronal loss in the hippocampus [2]. Moreover, patients with AD present mitochondrial dysfunction and metabolic changes, such as impaired glucose utilization in the brain (glucose hypometabolism) [5].

Mitochondrial dysfunction and a decline in respiratory chain function alter amyloid precursor protein (APP) processing, which leads to the production of the pathogenic amyloid-β fragments [6,7]. On the other hand, the reduced glucose uptake and inefficient glycolysis have been strongly associated with progressive cognitive deficiency [8], due to the downregulation of the glucose transporter GLUT1 in the brain of patients with AD [9]. Clinical studies have demonstrated an association between a high-glycemic diet and increased cerebral amyloid deposition in mice [10,11,12,13,14] and humans [15], suggesting that insulin resistance of brain tissue may contribute to the development of AD [16].

To date, there are only a few FDA approved drugs, such as acetylcholinesterase inhibitors and memantine. Drugs that regulate the activity of the neurotransmitters and partly ameliorate behavioral symptoms [17]. Another treatment option includes active and passive immunization, anti-aggregation drugs, γ- and β-secretases inhibitors [18]. Currently, there is no effective treatment to prevent the risk of AD development or modify its progress. Therefore, emerging results from preclinical and clinical studies show that change in dietary and lifestyle modifications may have a potential interest in the treatment of AD [19]. These recommendations include minimizing the intake of trans fat and saturated fats, dairy products and increased consumptions of vegetables, fruits, legumes (beans, peas, and lentils), and whole grains [19,20]. Moreover, various dietary patterns are suggested in order to reduce the neuropathological hallmarks of AD, including ketogenic diet (KD), caloric restriction (CR), the Mediterranean diet (MedDi), Dietary Approaches to Stop Hypertension (DASH), and Mediterranean-DASH diet Intervention for Neurological Delay (MIND) [20].

The ketogenic diet was initially established in the 1920s to be used in refractory epilepsy therapy [21,22]. To date, there are pieces of evidence showing that it has gained interest as a potential therapy for neurodegenerative disorders, such as AD [10,23], Parkinson’s disease [24], amyotrophic lateral sclerosis [25], and insulin resistance in type 2 diabetes [26]. Moreover, because of altered glucose metabolism, it may have anti-tumor effects, as well as, for example, in glaucoma [27], or gastric cancer [28]. Despite the growing number of evidence that dietary treatment works, the exact mechanism of its protective activity remains unknown.

This review summarizes the experimental and clinical data, which suggest that the ketogenic diet could be a potential therapy option for AD, due to its neuroprotective properties.

## 2. Etiopathogenesis of Alzheimer’s Disease

The etiology of AD remains not fully explained, but both genetic and environmental risk factors have been proposed to be involved. Thus, the etiopathogenesis of AD has been linked to hypometabolism [29,30], mitochondrial dysfunction [31], inflammation [32,33], and oxidative stress [21]. Some more cellular events associated with AD neuropathogenesis include impairment of calcium homeostasis and disturbed autophagy [32]. On the brain tissue level, neurons loss, brain atrophy, and cerebral amyloid angiopathy have to be mentioned [32]. In addition, the systems-level characteristic for AD involves the blood-brain barrier (BBB) abnormalities, brain arteries atherosclerosis, and brain hypoperfusion [32]. Moreover, genome-wide association studies (GWAS) have revealed that more than 20 genetic loci may be implicated with the risk of AD development [34]. The primary gene is the apolipoprotein E (ApoE), and the epsilon 4 (E4) variant of ApoE was found to increase the risk for AD generation [34]. Insulin resistance and type 2 diabetes mellitus are the essential risk factors of AD [3].

The neuropathological features of the AD brain include extracellular diffuse and senile amyloid plaques and intracellular neurofibrillary tangles. Amyloid plaques contain amyloid β peptides consisting of 38 to 43 amino acids generated by cleavage of neuronal cell membrane glycoprotein (APP) by β- and γ-secretases [32]. The main isoforms of Aβ have been distinguished: Aβ1-40 (90%) and Aβ1-42 (10%) [35]. β-secretase by cleaving the extracellular domain of APP and releasing the soluble N-terminal of APP into the extracellular space initiates the amyloidogenic pathway. Subsequently, the C-terminal of APP is cleaved by γ-secretase eventually yielding Aβ and APP intracellular domain (AICD) [35]. As a matter of fact, the non-amyloidogenic processing does not result in the production of Aβ, due to the cleavage of APP by α-secretase, leading to the release into the extracellular space of a soluble neuroprotective protein—sAPPα. Finally, γ-secretase cleaves the remaining the C-terminal fragment C83, yielding P3 and AICD. The increase in the concentration of Aβ leads to neurotoxicity and neurons loss. Interestingly, Aβ at brain lower concentrations seems to promote neurogenesis and plasticity, exert neurotrophic functions, influence calcium homeostasis, antioxidative processes, and redox sequestration of metal ions. Elevated generation of Aβ accompanied by its reduced clearance clearly results in the accumulation of Aβ and its subsequent neurotoxicity. The accumulated Aβ1-42 can undergo aggregation, which eventually leads to the formation of insoluble oligomers and fibrillary arrangement, the final step being senile amyloid plaques [36].

NFTs are composed of abnormally hyperphosphorylated tau protein, located within neurons [36]. The assembly and stabilization of microtubules requires tau protein, being crucial for cytoskeleton and transport of vesicles and organelles along the axons. Moreover, they play a role in the regulation of synaptic plasticity and synaptic function [37]. Under physiologic conditions, phosphorylation of tau protein by kinases is balanced by dephosphorylation by phosphatases, but the change in structure is observed when tau protein is hyperphosphorylated. The development of paired helical filaments (PHFs) and/or NFTs, causing destabilization of microtubules, as well as synaptic and neuronal injury [36].

## 3. Ketogenic Diet

The ketogenic diet assumes a very high-fat and low-carbohydrate diet, reducing carbohydrate to ≤10% of consumed energy. This restriction triggers a systemic shift from glucose metabolism toward the metabolism of fatty acids (FAs) yielding ketone bodies (KBs), such as acetoacetate (AcAc) and β-hydroxybutyrate (β-OHB) as substrates for energy [38]. Approximately 20% of basal metabolism for the adult brain is provided by the oxidation of 100–120 g of glucose over 24 h [39]. The KD provides sufficient protein for growth and development, but insufficient amounts of carbohydrates for the metabolic requirements [40]. Thus, energy is mostly derived from fat delivered in the diet and by the utilization of body fat [40]. The ketogenic diet is a biochemical model of fasting [41], which promotes organs to utilize KBs as the dominant fuel source to replace glucose for the central nervous system (CNS) [42].

Within hours of starting the diet, changes in plasma KBs, glucose, insulin, glucagon, and FAs levels are observed [43], which results in a drop in blood glucose concentration, as well as the insulin-to-glucagon ratio. An increased glucagon concentration is associated with the mobilization of glucose from its liver resources. Thus, the inhibition of glycogenesis and glucose reserves become insufficient for the fat oxidation process [44]. After 2–3 days of fasting, the primary source of energy is KBs, produced in the mitochondrial matrix of hepatocytes [45]. The higher level of KBs in the blood and their elimination via urine cause ketonemia and ketonuria [45]. Under physiological conditions, the blood concentration of KBs ranges from <0.3 mM, compared to glucose concentration ~4 mM, to 6 mM during prolonged fasting [46]. When KBs achieve concentrations above 4 mM, they become a source of energy for the CNS. In diabetic ketoacidosis, KBs may reach the level of 25 mM [39], resulting from an insulin deficiency with an increased glucose concentration (>300 mg/dL) and decreased blood pH (pH < 7.3), which may cause the death of the patient [45].

The KD allows ~90% of total calorie income from fat and much lower from protein (6%) and carbohydrate (4%) [21]. This may be achieved, due to a macronutrient ratio of 4:1 (4 g fat to every 1 g protein and carbohydrates) [21]. Thus, it includes replacing carbohydrates by fats in daily meals [41]. The most common KD form contains mainly long-chain fatty acids, although KD requires changes in eating habits, which is challenging to maintain, especially from a long-term perspective [44]. Therefore, a new form of KD was proposed. A diet based on medium-chain triglycerides (MCT) leads to similar effects by increasing the concentration of KBs in the blood, even if carbohydrates were present in the diet [44,47]. Another version of KD is the Atkins diet, in which carbohydrates are limited to 5% of energy in the diet [44].

As already mentioned, due to the restriction of glucose metabolism, KD requires to obtain energy from FAs of adipose tissue. Remarkably, the brain, due to its reduced ability to utilize FAs as an energy source, has to use KBs instead. KBs, through the mitochondrial β-oxidation of FAs yielding acetyl-CoA, are synthesized in the liver [7,48]. Some acetyl-CoA molecules remaining may be utilized in the Krebs cycle or to produce AcAc, further being converted spontaneously to acetone or β-OHB by β-OHB dehydrogenase (BDH) [7,48,49,50]. Later on, KBs enter the bloodstream and are available for brain, muscle, and heart, where they generate energy for cells in mitochondria [51]. β-OHB and AcAc can cross the BBB through proton-linked, monocarboxylic acid transporters, and provide an alternative substrate for the brain. Their expression is related to the level of ketosis [52]. During the long period of starvation, KBs may provide up to 70% of cerebral energy requirements [46]. When KBs are present at sufficient concentrations, they can maintain the basal (non-signaling) neuronal energy needs and up to ~50% of the activity-dependent oxidative neuronal requirements [53].

**Figure 1:**
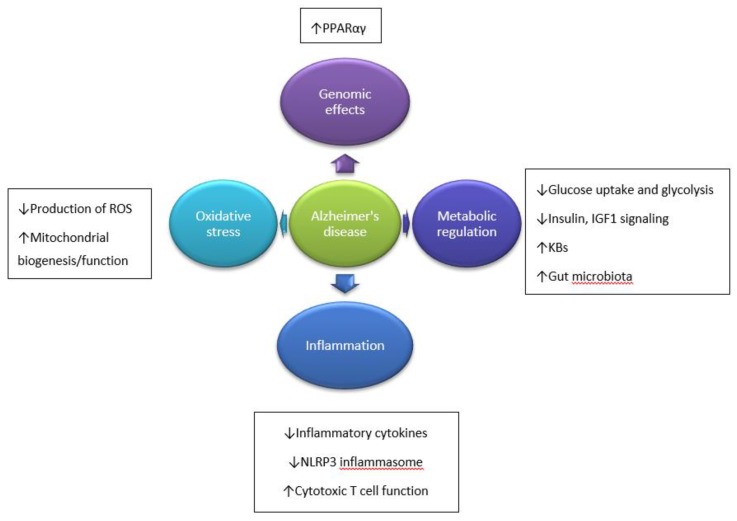
Hypothesized mechanisms through which ketogenic diet (KD) influence Alzheimer’s disease (AD) development. ↓—decreased; ↑—increased. Based on Reference [7].

Research studies evoked that KBs provide a more efficient energy source compared to glucose. They are metabolized faster than glucose and are able to bypass the glycolytic pathway by directly entering the Krebs cycle, whereas glucose needs to undergo glycolysis [7,46,54]. Because it leads to fatty acid-mediated activation of peroxisome proliferator-activated receptor α (PPARα), the glycolysis and FA are inhibited [50,55]. Thus, KBs reduce glycolytic ATP production and elevate ATP generation by mitochondrial oxidation [50], which enhances oxidative mitochondrial metabolism resulting in beneficial downstream metabolic changes. It includes the ketosis, higher serum fat levels, and lower serum glucose levels contributing to protection against neuronal loss by apoptosis and necrosis. Bough et al. [56] found that KD modulates the upregulation of hippocampal genes, which encode mitochondrial and energy metabolism enzymes [56]. Consequently, therapeutic ketosis can be considered as a form of metabolic therapy by providing alternative energy substrates. Through these metabolic changes, brain metabolism is improved, and ATP production in mitochondria is restored. Moreover, decreased reactive oxygen species (ROS) production, antioxidant effects, lower inflammatory response, and increased activity of neurotrophic factors are observed [7]. Another impact includes stabilization of the synaptic activity between neurons through increased levels of Krebs cycle intermediates, increased GABA-to-glutamate ratio, and activation of ATP-sensitive potassium channels [7]. Probable mechanisms underlying the beneficial influence of KD on AD development are presented in Figure 1.

### 3.1. The Impact of the Ketogenic Diet on Amyloid and Tau Protein

Defects in mitochondrial and respiratory chain function may alter APP processing, resulting in production neurotoxic Aβ [57]. The ketogenic diet could alleviate the effects of impaired glucose metabolism [8,58] by providing ketones as alternative metabolic substrates for the brain. Besides, this diet may help to reduce the deposition of amyloid plaques by reversing the Aβ(1–42) toxicity [58,59]. Studies suggest that KD may affect neuropathological and biochemical changes observed in AD. Rodents treated with the KD, exogenous β-OHB, and MCT display reduced brain Aβ levels, protection from amyloid-β toxicity, and improved mitochondrial function [10,30]. In the transgenic mice model of AD, it was observed that KD made soluble Aβ deposits level in their brain 25% less after only 40 days [60]. Also, in humans, this process may be determined by the presence or absence of the ApoE4 genotype; however, the presence of which is a risk factor for AD development [23,47].

Evidently, AD neuropathology is associated with aberrant hyperphosphorylation of tau protein. Mitochondrial dysfunction and decreased neuronal and glial mitochondrial metabolism follow in older people. The mitochondrial dysfunction results in diminished energy generation from the oxidation of glucose/pyruvate, and it can also increase Aβ accumulation and tau protein dysfunction. Consequently, the abnormal mitochondria could be characterized by an increased superoxide generation with subsequent oxidative injury, a decrease in oxidative phosphorylation, and finally resulting impairment of the mitochondrial electron transport chain [61].

### 3.2. The Impact of the Ketogenic Diet on Inflammation

Inflammation and oxidative stress are two essential factors recognized in the neuropathology of AD, underlying neurotoxic mechanisms leading to neuronal loss, which is present in the brain regions responsible for memory and cognitive processes [21,62]. It involves releasing proinflammatory cytokines, NO, and inhibition of neurotrophins, resulting in damage to surrounding tissues [62]. 

Because a great proportion of cells in the immune system (e.g., macrophages or monocytes) express abundant GPR109A, KD may actually affect neuroinflammatory mechanisms [63]. GPR109A, which was found in the brain tissue is, in fact, a G protein-coupled receptor known as hydroxy-carboxylic acid receptor 2 (HCA2) [63]. Moreover, the β-OHB may directly bind to HCA2, which is expressed on microglia [63], dendritic cells, and macrophages [64]. Its activation induces the neuroprotective subset of macrophages, which depend on PGD2 production by COX1 [64]. Consequently, neuroinflammation is reduced [63].

KD has also been proved to exert effects on inflammatory processes [65] by inhibiting the activation of the nuclear factor kappa-light-chain-enhancer of activated B cells (NF-kB). It results in the downregulation of COX2, and inducible nitric oxide synthase expression, associated with increased immune response [55]. Moreover, the activity of cytokines, such as IL-1b, IL-6, CCL2/MCP-1, TNF-α, is diminished [66]. Besides, peroxisome proliferator-activated receptor γ (PPARγ) can reduce the expression of NF-kB, therefore alleviating the neuronal damage caused by excitotoxicity of N-methyl-D-aspartate (NMDA) [67,68].

Moreover, the KD diet influences the anti-inflammatory action via activation of microglial cells [69], pro-apoptotic properties, and elevated concentrations of neuroprotective mediators, including neurotrophins {neurotrophin-3 (NT-3), brain-derived neurotrophic factor (BDNF) and glial cell line-derived neurotrophic factor (GDNF)}, and molecular chaperones (proteins preventing aggregation of polypeptides into potentially toxic molecules) [44,70].

Another mechanism of KD is the inhibition of histone deacetylases (HDACs), which play a role in altering chromatin structure, and accessibility [21]. β-OHB inhibits HDACs 1, 3, and 4 (class I and IIa) in vitro, leading to memory function improvement and synaptic plasticity [56,71]. Besides, ketones are able to inhibit the innate immune sensor NOD-like receptor 3 (NLRP3) inflammasome, which controls the activation of caspase-1, and the release of proinflammatory cytokines, such as IL-1β and IL-18 by limiting the K^+^ efflux from cells [42,50,72].

Also, it has been observed that β-OHB may revert the increased expression of inflammatory cytokines [73]. Lee et al. [74] have observed an elevated expression of cytokine interferon γ in the hippocampus of rats, which leads to protecting cells against excitotoxicity [74]. Ultimately, reducing inflammation could be one of the most crucial AD modifying effects of a KD.

### 3.3. The Impact of the Ketogenic Diet on Dementia

The main symptom of some neurodegenerative disorders is dementia, and it includes thinking difficulties, loss of memory, and obstacles in problem-solving. Progressive impairment of cognitive functions in AD patients was associated with a reduction in glucose uptake and metabolism [8], especially if genetic risk factors for AD or positive family history are present. Another possible mechanism is that lower glucose uptake in the brain may contribute to the development of AD neuropathology [45]. The study of Vanitallie [75] shows that an early disturbance in brain glucose metabolism can be detected before any measurable cognitive decline [75]. Moreover, it correlates with the downregulation of glucose transporter GLUT1 in people with AD [76]. It is observed that a high-glycemic diet is associated with increased insulin resistance and a higher risk of AD development [15]. Few studies have demonstrated that supplementation with MCT and KD improves cognitive performance [23,47,77,78,79,80,81,82].

The hypometabolism in brain tissue has been referred to indicate a risk for the development of dementia in the future [83], following chronic brain energy deprivation, then impairment of neuronal function, and in later stages decline in glucose demand along with the progression difficulties of cognitive performance [84]. In addition, progressive dementia was correlated with reduced blood flow and oxygen consumption in the brain [84]. 

The altered glucose metabolism and mitochondrial function may result from the accumulation of advanced glycation end products (AGEs) [85]. Although the presence of AGEs in cells and tissues is a characteristic feature of the aging process, it may be enhanced in AD pathology. Also, AGEs molecules can be found in amyloid plaques and neurofibrillary tangles resulting from oxidative stress, protein crosslinking, and neurons cell loss. To sum up, the reduced glycemia could advance these pathophysiological features in AD [45].

### 3.4. The Impact of the Ketogenic Diet on Neurodegeneration

AD is associated with energy imbalance caused by impaired glucose transport and metabolism and mitochondrial dysfunction. Energy deficiency may be observed in different brain structures, especially in the hippocampus [29]. Within the AD neuropathology, there is a shift in brain metabolism, which results in diminished cerebral glucose utilization [86]. On the other hand, increased ketogenesis is observed during the aging process [86].

Mitochondrial dysfunction and oxidative stress play a significant role in neurodegeneration. Both processes are known to generate higher concentrations of ROS, which are harmful to all cellular macromolecules, including nucleic acid, lipid, and protein damage [87]. Therefore, KD may provide neuroprotective benefit by improving mitochondrial function through biochemical changes resulting from glycolysis inhibition and increased KBs formation. It is observed that metabolic ketosis may decrease ROS production improving mitochondrial respiration and bypassing complex 1 dysfunction [48].

Moreover, KD modulates the ratio between the oxidized and reduced forms of nicotinamide adenine dinucleotide (NAD+/NADH). An increased NAD+/NADH ratio plays a role in protection against ROS and improves redox reactions, mitochondrial biogenesis, and cellular respiration, which stabilizes synaptic action [56,88]. A significant increase in the NAD+/NADH ratio was found in the brain cortex and hippocampus of KD-fed rats after two days [54]. After all, it induces the gene expression via sirtuin 1 (SIRT1), a type 3 histone deacetylase [89], involved in different processes related to deacetylating histone and non-histone targets [21,90]. Also, SIRT1 may limit the oxidative stress by improving the synthesis of heat shock proteins [91], promoting DNA repairing activity of forkhead transcription factor (FOXO) and protein p53 [92], and deacetylation of nuclear factor erythroid 2-related factor 2 (Nrf2), the primary inducer of detoxification genes [93]. In addition, the increased activation of Nrf2 results from the increased production of hydrogen peroxide in the mitochondria, and elevated level of lipid peroxidation product—4-hydroxy-2-nonenal (4-HNE) [94]. In addition, Nrf2 is capable of inducing glutathione reductase, peroxiredoxin and thioredoxin, the primary enzymes responsible for the regeneration of the active form of endogenous antioxidant agents [95], followed by the expression of heme oxygenase-1 (HO-1), an antioxidant protein considered to be one of the key molecules in neuroprotection against oxidative stress [67].

Therefore, KD increases the efficiency of the electron transport chain through the increased expression of uncoupling proteins (UCP), and their activity in the hippocampus [96] by blocking voltage-gated sodium and calcium channels, and regulates the membrane receptors in neurons [97]. Thus, mitochondrial energy reserves may be increased [70,96]. UCP moderates the mitochondrial membrane potential and declines the production of ROS and reactive nitrogen species (RNS) [98].

Moreover, KD increases levels of superoxide dismutase 2 (SOD2), mitochondrial mass, and regulators, such as SIRT1 and mitochondrial fission 1 protein (FIS1); thus, appears to upregulate γ-aminobutyric acid (GABA) A receptor subunits α1, and downregulate NMDA receptor subunits NR2A/B [87].

In addition, KBs may regulate the homeostatic status of mitochondria by changing the calcium-induced membrane permeability transition (mPT) and inhibit opening the pores [42,99]. Also, the selected polyunsaturated fatty acids (PUFAs), such as eicosapentaenoic acid, arachidonic acid, or docosahexaenoic acid may promote excitability of neuron-cell membranes by suppressed ROS production, decreased inflammatory mediators, and blocking voltage-gated sodium and calcium channels [100]. In addition, KD increases glutathione levels and glutathione peroxidase (GSH-Px) activity in the hippocampus [101], which is the main enzyme affecting the formation of ROS [97].

The potential mechanism of action may be through modulation of intracellular signaling pathways, including the mammalian target of rapamycin (mTOR). Studies show that KD decreases insulin levels and reduces the phosphorylation of Akt and S6, which results in diminished mTOR activation [42,102]. KD also leads to elevated brain ATP and phosphocreatine concentrations, and stimulates mitochondrial biogenesis, which may be interpreted in terms of enhanced metabolic efficiency [56]. Finally, neuronal cells can be considered to have improved resistance and adaptability to stress and metabolic challenges [50,56].

### 3.5. Adverse Effects of the Ketogenic Diet

Data on the adverse effects of KD administration is limited in the adult population, but some effects are predictable, such as hypoglycemia and dehydration. Other side effects are less common and present following long-term treatment.

Previously, KBs were considered toxic resulting from the association of therapeutic ketosis with diabetic ketoacidosis, which results in ketone concentrations higher than 20 mM, which can be reversed with insulin administration [103]. Hyperketonemia resulting from insulin deficiency, in severe cases, may lead to severe acidosis, and even death of the individuals [45,104].

The adverse effects frequently reported by patients with epilepsy on KD are gastrointestinal effects, weight loss, and transient hyperlipidemia [42]. Gastrointestinal side effects can include constipation, nausea, vomiting, and lower appetite [42,105]. Weight loss may be a welcomed effect, especially in an obese patient, but it should be regulated and monitored. In addition, the change in lipid profile, such as fasting total serum cholesterol, triglycerides, and low-density lipoprotein (LDL) cholesterol is increased at the beginning of the KD treatment then it normalizes (after ~1 year) [106]. Moreover, dehydration, hepatitis, pancreatitis, hypoglycemia, hyperuricemia, hypertransaminemia, hypomagnesemia, and hyponatremia are among the adverse effects of the KD [44,105]. On the other hand, prolonged KD may cause enhanced atherosclerosis, cardiomyopathy, nephrolithiasis, impaired hepatic functions, neuropathy of the optic nerve, anemia, reduction of mineral bone density, and deficiencies of vitamins and mineral components [44].

Chronic KD treatment may cause disturbances in catabolism and reduced synthesis of functional proteins (membrane proteins, enzymes, etc.). Considering the loss of appetite and lower organoleptic attractiveness, it would be difficult to achieve an appropriate supply of protein and energy in patients on the KD, because any energetic deficiency or insufficient protein intake may have severe consequences for health [44,81]. Any significant adverse effects were not observed in 83 obese patients when the KD was administered for 24 weeks [107]. Additionally, in patients with AD, KD may significantly affect food consumption via disturbances in the senses of smell and taste, neurological symptoms, such as apraxia, dysphagia, and behavioral disturbances during eating [44].

## 4. The Mechanism of Neuroprotective Action of the Ketogenic Diet

**Figure 2:**
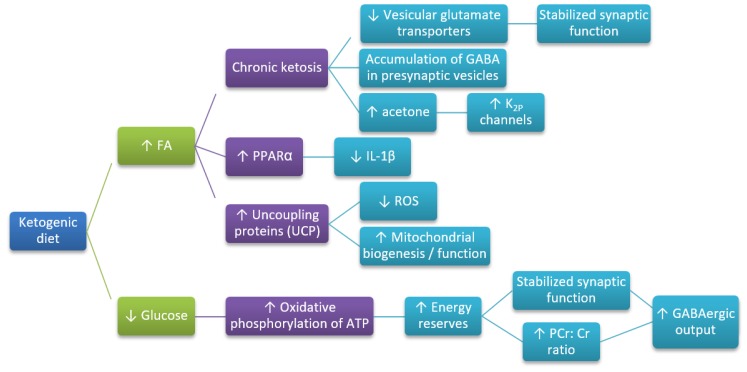
Hypothetical pathways leading to the neuroprotective action of KD (based on References [50,108]). FA—fatty acids; GABA—γ-aminobutyric acid; PCr:Cr—phosphocreatine:creatine ratio; ROS—reactive oxygen species; UCP—uncoupling proteins; increase (↑) or decrease (↓)—arrows indicate the direction of the relationship between variables.

The mechanism of neuroprotective action of KD (Figure 2) remains not fully understood, but several studies show that KBs influence neurons loss at three different levels, such as (i) metabolic level; (ii) signaling level; (iii) epigenetic level. Numerous mechanisms have been established through which the KD may contribute to the neuroprotective activity. The effectiveness of KD was checked in a limited number of clinical trials. However, there are studies in vitro or in animal models assessing the underlying biological mechanisms. The main goal of AD treatment is primary the prevention of specific neuropathological damages, associated with amyloid plaques and neurofibrillary tangles accumulation. Another focus of research includes brain metabolism dysregulation, neuronal signaling, and mitochondrial homeostasis. The activity of the ketogenic diet is associated with the decrease of the inflammatory response and the oxidative damage. Reduced blood glucose level and elevated concentration of KBs are the main characteristic features of KD treatment.

The effects of KBs are associated with an increased level of acetone, which may activate K_2P_ channels to hyperpolarize neurons and limit neuronal excitability [50,108]. Also, KBs affect the altering metabolism of neurotransmitters, such as glutamate and GABA in the brain. Moreover, the activity of brain-specific UCPs is increased, which reduces ROS generation by mitochondrial complex I, modulates dysfunction of neurons, and aftereffect neurodegeneration [50,108]. Ketosis would induce the expression of UCPs and coordinately upregulate several dozen genes related to oxidative energy metabolism by acting via the nuclear transcription factor PPARα and its co-activator peroxisome proliferator-activated receptor γ coactivator-1 (PGC-1α) [50,108]. Ketosis has been shown to stimulate mitochondrial biogenesis, thus leading to increased generation of ATP and enhanced energy reserves, which is known to stabilize synaptic activity. Probably, the elevated phosphocreatine:creatine (PCr:Cr) energy-reserve ratio may potentiate GABAergic output, very likely associated with the ketosis-triggered increased GABA synthesis [50,108]. During KD, the decreased glucose availability, with accompanying elevated FAs, is suggested to reduce glycolytic flux. Consequently, it would further be feedback inhibited by elevated concentrations of ATP and citrate formed during KD treatment. Thus, K_2P_ channels would be activated [50,108]. Moreover, KD involves changes in microbiome followed by involvement of the gut-brain axis [109]. Olson et al. [109] present that KD alters the gut microbiota required for protection against several kinds of seizures [109].

## 5. Preclinical and Clinical Studies

Epidemiological observations provides evidence that a diet rich in saturated fatty acids may elevate the risk of AD [19]. Also, transgenic mice fed with a fat-rich diet may exhibit accelerated cognitive disturbances, due to enhanced oxidative stress, systemic inflammation, and increased neuronal death, due to apoptosis [110,111]. At the same time, the increasing number of animal studies and clinical trials on humans show the benefits of KD treatment in AD. The summary of the preclinical and clinical studies with their main findings is presented in Table 1 and Table 2.

### 5.1. Preclinical Studies

In the transgenic model of AD, mice fed with a KD exhibited better mitochondrial function, decreased Aβ accumulation, and oxidative stress when compared to healthy controls [10]. Auwera et al. [10] reported that KD low in carbohydrates and rich in saturated fats reduced the level of Aβ in transgenic mice, expressing a human APP gene with London mutation (APP/V717I). This particular mutation results in significant levels of soluble brain Aβ (as early as three months of age) and extensive plaque formation by 12–14 months [10,115]. Moreover, in transgenic mouse models, high-fat diets increase the deposition of Aβ peptides [116,117]. Exposure to a KD for 43 days resulted in a 25% reduction in soluble Aβ(1–40) and Aβ(1–42) in brain homogenates, but did not affect performance on the object recognition task [10]. Another study in young, healthy mice shows that KD may influence the brain vascular function, improve metabolic profile (decreased blood glucose and increased KBs levels), and alter the gut microbiome [118].

Various mouse models of AD (APP/PS1, mouse models of Aβ deposition, carrying mutations in APP and/or presenilin 1, and Tg4510, mouse model as a model of tau deposition) under KD (low carbohydrate, MCT-rich diet) had elevated KBs level and reduced glucose levels in the bloodstream [11,12]. In this study, any reduction in Aβ or tau protein accumulation was not observed; however, an improved motor performance on the Rotarod apparatus was present [11,12]. Kashiwaya et al. [30] demonstrated that long-term (8 months) feeding of a ketone ester in middle-aged mice (8.5 months old) improved their cognition and ameliorated Aβ and tau protein pathology [30]. KD may mitigate apoptosis by inhibition of kainic acid-induced accumulation of the protein clusterin, thought to influence apoptotic signaling [59]. Moreover, KD and β-OHB administration may protect dopaminergic neurons from degeneration [59].

In aged rats, the administration of KD for over three weeks improved learning skills and memory. It was associated with increased angiogenesis and capillary density suggesting that KD may support cognition through improved vascular function [119]. Moreover, pretreatment with MCT showed reduced Aβ deposition in rat cortical neurons affecting glucose metabolism via activation of signaling pathways [120]. Study on senile dogs also indicates that the MCT diet may improve mitochondrial function by control of the oxidation process, and decrease Aβ concentration in the brain [121].

### 5.2. Clinical Studies

In the first randomized controlled trial, 20 patients with MCI or AD received a single oral dose of MCT [23]. Reger et al. [23] found that the acute administration of MCT improves memory in AD patients [23]. Further, the degree of memory performance was positively correlated with a β-OHB concentration in plasma, produced by oxidation of the MCT. AD patients have been demonstrated to possess defects in brain glucose metabolism, which may be caused by neurotoxic Aβs or disturbed lipid homeostasis [9]. Moreover, the apolipoprotein E4 (ApoE4) genotype has an impact on the outcome of the KD treatment. Patients without ApoE4(−) allele exhibited improved short-term cognitive performance on a screening tool of memory, language, attention, and praxis [23]. Moreover, the cognitive effects of long-term elevation of β-OHB concentrations may speak for the feasibility and efficacy of MCT as a novel treatment strategy [23].

Reger et al. [23] and Henderson et al. [47] compared the influence of an MCT on memory and cognition in double-blind trials controlled with placebo. Both trials clearly indicated that elevated serum β-OHB levels caused improvements in cognitive function and memory. Further sub-analysis in the evaluated cohorts was carried out to assess for the ApoE4 status of the patients. ApoE4(+) patients have a mutation associated with an increased risk of AD development. In both studies, ApoE4(+) was associated with a reduced response to KD.

Besides, Krikorian et al. [77] compared a low carbohydrate diet with a high carbohydrate diet in 23 adult patients with MCI treated for over six weeks. The low carbohydrate diet showed better verbal memory performance, positively correlated with KBs levels in the carbohydrate-restricted group. Nevertheless, no significant difference in cognitive function between the groups was evident [77]. The authors conclude that even short-term use of a low-carbohydrate diet could have a beneficial impact on memory function in older adults with an increased risk for AD. However, the mechanism could be associated with reduced inflammation and enhanced energy metabolism.

In the case study by Newport et al. [78], effects on cognitive functions were evaluated in an adult AD patient using ketone monoester (R)-3-hydroxybutyl-(R)-3-hydroxybutyrate supplementation for 20 months to stimulate ketosis [78]. Actually, the patient was improved in terms of mood, affect, self-care, cognitive, and daily activity performance [78]. Another three studies were performed in patients with MCI or mild to moderate AD. Using at least three-month treatment protocols (two randomized studies of MCT or a ketogenic product compared to placebo for three to six months and one observational study administering KD over three months), it was reported that the cognitive benefit of KD treatment was highest in ApoE4(−) patients [79]. In the observational study, it was limited to ApoE4(−) patients with mild AD [80]. These clinical studies propose that KD may improve cognitive function in patients with AD by inducing metabolic ketosis. However, the ApoE4(+) genotype and the degree of disease progression have an impact on body response to metabolic ketosis.

In addition, increased ketone intake, as shown by imaging of PET 11C acetoacetate in the brain before and after treatment, was evident in patients with mild to moderate AD after MCT supplementation within one month. It can be suggested that ketones from MCT can compensate for brain tissue glucose deficit in patients with AD [84]. The clinical evidence seems to support the hypothesis that KDs may improve cognition in AD patients. However, data may be strictly associated with the stage of AD, its progression, and the ApoE4 genotype, which may determine response to dietary administration [84].

The additional study was a single-arm pilot trial in 15 patients with mild-moderate AD, where MCT-supplemented ≥1:1 ratio KD was administered for three months. It showed cognitive function improvement in 9 out of 10 patients who completed the study and achieved ketosis [81]. Moreover, in another study called the Ketogenic Diet Retention and Feasibility Trial, MCT-supplemented KD was provided to 15 AD patients (~70% of energy as fat). When ketosis was achieved, the ADAS-cog test was significantly improved during the KD [81].

In a recent study, MCT were administered to 20 Japanese patients with mild to moderate AD over 12 weeks [82]. After 120 min of intake, KBs level was increased, then after eight weeks, the patients demonstrated significant improvement in their immediate and delayed memory tests compared to their baseline score [82].

## 6. Conclusions

The perspective of the use of KD in various diseases has been growing recently. Abnormal glucose metabolism uptake, diminished mitochondrial-associated brain energy metabolism, changes in neurotransmitter release, and increased inflammatory response are the key pathophysiological metabolic alterations observed in AD. Furthermore, KD may modulate a broad array of metabolic and signaling changes underlying the pathophysiology of neurodegenerative disorders.

Based on the limited animal studies and clinical trials, KD has beneficial effects for enhancing mitochondrial function and cellular metabolism. It is associated with improved cognitive performance in elderly adults with AD. The improvement of the cognitive outcomes depends on the level and duration of ketosis. The best results of KD treatment are expected in early presymptomatic stages of AD. However, it requires a practical diagnostic approach.

The future research should explore the exact mechanism (s) of action of KD that underlie the neurodegenerative disorders to restore abnormal glucose and energy metabolism in animal models, as well as in patients with different diseases. Also, further studies are necessary for the long-term effects of KD for nutritional status, general well-being, and the progress of AD in patients. However, this novel metabolic treatment seems to be intriguing and deserves further clinical investigations in the progress of AD.

## Figures and Tables

**Table 1:** Main preclinical evaluations of KD treatment in AD.

Preclinical Studies		
Model	Main Findings	Ref.
• Hippocampi of juvenile mice	• Improvement of mitochondrial function • Decreased ROS production • Increased cerebral ATP concentrations	[96]
• KD in rats	• Reduced insulin levels • Reduced phosphorylation of Akt and S6 • Decreased mTOR activation	[102]
• KD in rats	• Increased lipid peroxidation product 4-hydroxy-2-nonenal (4-HNE) levels • Increased activation of Nrf2	[112]
• In vitro models	• Inhibition of histone deacetylases (HDACs) • Increased transcriptional activity of PPAR-γ	[113]
• KD in the APP/V717I transgenic mouse model of AD	• Better mitochondrial function • Reduced oxidative stress • Reduced Aβ deposition	[10]
• KD in the APP/PS1 mouse model of AD	• Improvement of motor function • Improvement in energy metabolism • Reduced Aβ deposition	[11] [12]
• KD in the Tg4510 mouse model of AD	• Improvement of motor function • Improvement in energy metabolism • Reduced Aβ deposition	[13] [14]
• Administration of ketone ester in middle-aged mice (8.5 months old) over eight months	• Improvement of cognitive function • Ameliorated Aβ and tau protein pathology	[30]

AD—Alzheimer’s disease; APP—amyloid precursor protein; Aβ—amyloid β-peptide; KD—ketogenic diet; PPAR-γ—peroxisome proliferator-activated receptor γ; PS1—presenilin 1; ROS—reactive oxygen species; β-OHB—β-hydroxybutyrate.

**Table 2:** Main clinical evaluations of KD treatment in AD.

Clinical Evaluation			
Type of Study	Protocol	Main Findings	Ref.
Double-blind placebo-controlled trial	• 20 adult patients with AD or MCI • Administration of MCT	• Significant increases in β-OHB levels moderated by ApoE4 genotype (greater for ApoE4(+) compared to ApoE4(−)) • Improvement of memory and cognitive function in the ADAS-cog test compared to placebo • Patients ApoE4(+) were less responsive to KD compared to ApoE4(−)	[23]
Randomized, Double-blind, Placebo-controlled multicenter trial	• 152 adult patients with mild to moderate AD • Administration of AC-1202 over 90 days	• AC-1202 significantly increases a β-OHB level resulted in • Significant improvement in the ADAS-cog test compared to placebo after 45 and 90 days of treatment • Reduced response to AC-1202 in ApoE4(+) patients compared to ApoE4(−)	[47]
Other clinical study	• 23 adult patients with MCI • Administration of high carbohydrate or very low carbohydrate diet over six weeks	• Significant improvement in verbal memory performance for the low carbohydrate subjects • Reductions in weight, waist circumference, fasting glucose, and insulin in the low carbohydrate group • KBs levels positively correlated with memory performance	[77]
Singe-patient case study	• One adult patient with early-onset AD • Administration of KME over 20 months	• Improved markedly in mood, affect, self-care, and daily activities • Improved cognitive performance • KME-induced hyperketonemia seems robust, convenient, and safe	[78]
Pilot and feasibility, randomized, double-blind placebo-controlled parallel trial	• Six adult patients with MCI • Administration of 56 g/day of MCT over 24 weeks	• Increased β-OHB levels • Improvement of memory in mild AD and ApoE4(−)	[79]
Prospective, open-label, observational study	• 22 adult patients with mild-to-moderate AD • Administration of a ketogenic meal “Axona” (40 g of powder containing 20 g of caprylic triglycerides) over 90 days	• No improvement in cognitive performance, even in ApoE4(−) patients	[80]
Single-arm pilot trial Ketogenic Diet Retention and Feasibility Trial (KDRAFT)	• Fifteen adult patients with mild-to-moderate AD using an MCT-supplemented ≥1:1 ratio KD for three months (a very high-fat ketogenic diet (VHF-KD))	• Increased β-OHB levels • Improvement in ADAS-cog in 9 out of 10 patients who completed the study and achieved ketosis	[81]
Other clinical study	• 19 adult patients • Administration of MCT-supplemented ketogenic meal (Ketonformula^®^) containing 20 g of MCT	• Increased β-OHB levels •Improvement of cognitive performance • Positive effects on visual attention, working memory, and performing tasks in non-demented patients	[82]
Double-blinded, placebo-controlled, randomized clinical trial	• 16 adult patients with mild-to-moderate AD • Administration of caprylidene over 45 days	• Increased cerebral blood flow in patients ApoE4(−)	[114]

AD—Alzheimer’s disease; ADAS-cog—Alzheimer’s Disease Assessment Scale-Cognitive Subscale; ApoE4—apolipoprotein E4; KBs—ketone bodies; KD—ketogenic diet; KME—ketone monoester; MCI—mild cognitive impairment; MCT—medium-chain triglycerides; β-OHB—β-hydroxybutyrate.

## Abbreviations

4-HNE	4-hydroxy-2-nonenal
AcAc	acetoacetate
AD	Alzheimer’s disease
LD	linear dichroism
ADAS-cog	Alzheimer’s Disease Assessment Scale-Cognitive Subscale
AGEs	advanced glycation end products
AICD	APP intracellular domain
ApoE4	apolipoprotein E4
APP	amyloid precursor protein
Aβ	amyloid β-peptide
BBB	blood-brain barrier
BDH	β-OHB dehydrogenase
CNS	central nervous system
CR	caloric restriction
DASH	Dietary Approaches to Stop Hypertension
FAs	fatty acids
FIS1	fission 1 protein (FIS1)
FOXO	forkhead transcription factor
GABA	γ-aminobutyric acid
GSH-Px	glutathione peroxidase
GWAS	genome-wide association studies
HDACs	histone deacetylases
HO-1	heme oxygenase-1 (HO-1)
KBs	ketone bodies
KD	ketogenic diet
LDL	low-density lipoprotein cholesterol
MCI	mild cognitive impairment
MCT	medium-chain triglycerides
MedDi	Mediterranean diet
MIND	Mediterranean-DASH diet Intervention for Neurological Delay
mPT	membrane permeability transition
mTOR	mammalian target of rapamycin
NF-kB	nuclear factor kappa-light-chain-enhancer of activated B cells
NFTs	neurofibrillary tangles
NLRP3	NOD-like receptor 3 inflammasome
NMDA	N-methyl-D-aspartate
Nrf2	nuclear factor erythroid 2-related factor 2
PCr:Cr	phosphocreatine: Creatine ratio
PGC-1α	peroxisome proliferator-activated receptor γ coactivator-1
PHFs	paired helical filaments
PPARα	peroxisome proliferator-activated receptor α
PPARγ	peroxisome proliferator-activated receptor γ
PUFAs	polyunsaturated fatty acids
ROS	reactive oxygen species
SIRT1	sirtuin 1
SOD2	superoxide dismutase 2
UCP	uncoupling proteins
β-OHB	β-hydroxybutyrate
